# Future Heat Waves in Different European Capitals Based on Climate Change Indicators

**DOI:** 10.3390/ijerph16203959

**Published:** 2019-10-17

**Authors:** Jürgen Junk, Klaus Goergen, Andreas Krein

**Affiliations:** 1 Environmental Research and Innovation, Luxembourg Institute of Science and Technology, 4422 Luxembourg, Luxembourg; andreas.krein@list.lu; 2 Institute of Bio- and Geosciences (IBG-3, Agrosphere) Research Centre, 52428 Jülich, Germany; k.goergen@fz-juelich.de; 3 Centre for High-Performance Scientific Computing in Terrestrial Systems, Geoverbund ABC/J, 52428 Jülich, Germany

**Keywords:** climate change indices, health risks, heat waves, RCP4.5, RCP8.5, regional climate projections

## Abstract

Changes in the frequency and intensity of heat waves have shown substantial negative impacts on public health. At the same time, climate change towards increasing air temperatures throughout Europe will foster such extreme events, leading to the population being more exposed to them and societies becoming more vulnerable. Based on two climate change scenarios (Representative Concentration Pathway 4.5 and 8.5) we analysed the frequency and intensity of heat waves for three capital cities in Europe representing a North–South transect (London, Luxembourg, Rome). We used indices proposed by the Expert Team on Sector-Specific Climate Indices of the World Meteorological Organization to analyze the number of heat waves, the number of days that contribute to heat waves, the length of the longest heat waves, as well as the mean temperature during heat waves. The threshold for the definition of heat waves is calculated based on a reference period of 30 years for each of the three cities, allowing for a direct comparison of the projected changes between the cities. Changes in the projected air temperature between a reference period (1971–2000) and three future periods (2001–2030 near future, 2031–2060 middle future, and 2061–2090 far future) are statistically significant for all three cities and both emission scenarios. Considerable similarities could be identified for the different heat wave indices. This directly affects the risk of the exposed population and might also negatively influence food security and water supply.

## 1. Introduction

The Intergovernmental Panel on Climate Change (IPCC) concludes in its Fifth Assessment Report (AR5) that the mean global near-surface air temperature showed a warming of 0.85 °C for the period from 1880 to 2012 and a warming of the climate system is unequivocal [1]. In the Special Report on Extremes (IPCC SREX), the IPCC highlights three possible changes in extreme events due to elevated air temperature: (a) increases in the mean temperature, (b) increases in the variability and (c) changes in the symmetry of the probability distribution [2].

June 2019 was the hottest month since 1880 worldwide with air temperatures of 3 °C above the average for Europe and 1 °C globally [3]. Even though it is difficult to attribute such a heat wave to climate change, those extreme events are projected to become more frequent as greenhouse gas concentrations are increasing [4,5]. Even small changes in the annual mean air temperature can result in large changes on the severity of such extreme events [6]. Changes in the frequency and intensity of heat waves (HWs) will have profound impacts on the natural environment and the human society [4]. The 2018 report in the Lancet entitled “Countdown on health and climate change: shaping the health of nations for centuries to come”, gives a very clear overview of the environmental changes already observed and their projected health impacts [7]. In 2017, compared to 2000, 157 million more people were exposed to heat waves worldwide. For the same year, 153 billion hours of labour were lost due to HWs, resulting in US $ 326 billion in economic losses [7]. Extremely high air temperatures that last for longer periods can lead to heat stroke, heart failure and inhibition to sustain physical activities [8,9,10,11]. High correlations between HW and mortality due to heart diseases could be observed especially for risk groups like minors, elderly people or persons with already existing illnesses [12,13].

In order to define adequate adaption and mitigation measures to reduce both the health risk and the negative economic impacts of HW, knowledge of the future frequency and intensity of such events is crucial. A multi model ensemble of transient regional climate change projections was retrieved from the public data archive of the Coordinated Regional Climate Downscaling Experiment (CORDEX). The same set of ensemble members (*n* = 13) for two different climate change scenarios (Representative Concentration Pathway 4.5 and 8.5) were used to analyse past and future HW characteristics. A time series was created, covering the period from January 1971 to December 2091, consisting of daily minimum and maximum air temperatures. 

Climate projections based on nested regional climate model approaches show increases in the frequency, intensity and duration of extreme temperature events [2,14]. One of the major problems when analysing HWs and their impacts on society, is the definition of HWs. To date, there is no universally accepted definition of HWs. In general, an HW is a prolonged period of consecutive days with extremely high air temperatures for a specific region [14], taking into account that average conditions in one region, e.g., the Mediterranean, would be regarded as extreme conditions in another region, like Northern Europe. In order to make such comparisons possible across different countries, the use of individual threshold values is recommended to define HWs derived from site-specific data sets.

The World Meteorological Organization (WMO) Commission for Climatology (CCI) Expert Team on Sector-specific Climate Indices (ET-SCI) developed an internationally coordinated set of core climate indices in order to detect changes in climate extremes [13,14]. The general purpose of these indices is to extract useful information from a large data set of atmospheric variables. These indices are based on daily values of minimum and maximum air temperatures as well as daily precipitation totals. They have been widely used to detect and attribute climate change signals in historical data sets [15,16,17], climate change projections [18,19,20], and of HW investigations [21,22,23]. These indices—relevant for HW—are used to analyse the projected changes in the heat stress level for three capital cities along a latitudinal transect across Europe.

## 2. Materials and Methods 

### 2.1. Climate Change Projections

For the calculation of the climate change indices, long, simulation-based time series of meteorological data are necessary. We used time series derived from a multi-model ensemble of climate change projections from the Coordinated Regional Climate Downscaling Experiment (CORDEX) project of the World Climate Research Programme (WCRP). The regional climate model (RCM) data are based on simulations from the EURO-CORDEX initiative. This is the European branch of the CORDEX project. Data is made available on the data nodes of the Earth System Grid Federation (ESGF) model data dissemination system [15]. 

One of the EURO-CORDEX goals is the provisioning of regional climate change information [16] through a dynamical downscaling of the Global Climate Models (GCM) of the Coupled Model Intercomparison Project (CMIP5) [17] for Representative Concentration Pathways (RCPs) RCP2.6, RCP4.5, and RCP8.5 [18], based on a coordinated multi-model, multi-physics experiment and providing transient model simulations from 1950 to 2100. Among the multiple CORDEX publications a basic analysis of climate change in the EURO-CORDEX ensemble is already available [16] as well as an overview evaluation of the ensemble with respect to observations [19]. Additionally, the added value of the higher EUR-11 (about 12 km) EURO-CORDEX resolution versus the standard EUR-44 (about 48 km) has been already analysed [20]. 

For this study, time series of daily maximum and minimum air temperature for London (51°30’ N 0°5’ E), Luxembourg (49°36’ N 6°7’ E), and Rome (41°54’ N 12°30’ E) were extracted from a locally stored partial copy of the EURO-CORDEX data repository. Taking into account data availability at the time of retrieval in November 2018, 13 matching ensemble members (Table 1), i.e. the same RCM with the same configuration, run by the same modelling group, were available overall from January 1971 to December 2090, for RCP4.5 and RCP8.5 at a high spatial resolution of about 12 km. All EURO-CORDEX RCMs utilized are driven by r1i1p1 GCM ensemble members. We refrained from including the RCP2.6 scenario ensemble members in our study, because for that scenario only four ensemble members were available in the archive. As it is only possible to compare different scenarios when the same ensemble members were used for the analysis, and including RCP2.6 would have reduced the size of the ensemble substantially.

The time series were extracted using a bilinear resampling algorithm (using the Climate Data Operators v1.8.1 software, https://code.mpimet.mpg.de/projects/cdo) from the EUR-11 RCM model grid; i.e., data from the four closest points to the city centre locations, representing an area of about 252 km^2^, were considered and averaged to daily spatial means with a linear distance weighing. Because of the low altitude of the cities no height correction was applied to account for altitudinal differences between the city centres and the actual RCM grid point altitudes. Also, because for this study relative differences between the future climate projections and the historical time spans are considered, a bias adjustment was not applied [21]. Unfortunately, not all the models use the same calendar. In model M13 leap years were not taken into account and in model M5 the first day (01.01.1971) was missing. Gaps due to leap years were filled via linear interpolation and the missing values for 1. January in M5 were replaced by the values of 2. January. No other gaps or missing values were detected in the time series of the air temperature data.

### 2.2. Definition of Indices

The open source R software package ClimPACT2 (https://github.com/ARCCSS-extremes/climpact2) was used to calculate indices related to heat waves on a yearly basis, namely the warm spell duration indicator, number of HW, number of days that contribute to an HW, duration of the longest HW, as well as the average temperature across all HWs within a year (Table 2). 

The definition of heat waves is based on three different threshold criteria: (a) the 90th percentile of the minimum daily temperature, (b) the 90th percentile of the maximum daily temperature, and (c) the Excess Heat Factor, all of which were derived from the 30-year reference period 1971–2000. If any of these thresholds were exceeded for at least three days, this period was considered an HW. A more detailed definition is given by [14].

After the identification of the HWs, those were analysed in more detail, based on the 4 indices HW-D, HW-F, HW-M and HW-N described in Table 2. In addition, the warm spell duration indicator (WSDI) was calculated, defined by a span of at least six days where the 90th percentile of the maximum temperature threshold taken from the reference period was exceeded.

### 2.3. Statistical Methods

The ClimPACT2 tool includes a test to detect outliers in the air temperature data time series. The criterion for detecting outliers can be defined by the user and is a certain number of standard deviation. In our study, we used the default value of 4 standard deviations to detect outliers. No outliers were detected in the input data sets. In addition, simple checks were performed, e.g., for cases where the maximum air temperature was below the minimum air temperature.

The results of all calculated indices, as well as the original time series, were further analysed with SigmaPlot Ver.10 from Systat Software, Inc. The time series of the indices were grouped into four 30-year time spans (1971–2000 reference period, 2001–2030 near future, 2031–2060 middle future, 2061–2090 far future). The three future time spans (near, middle and far future) were then tested for significant differences (*p* < 0.001) against the reference time span 1971–2000 with the Kruskal-Wallis Analysis of Variance (ANOVA) on Ranks. 

## 3. Results

**Figure 1:**
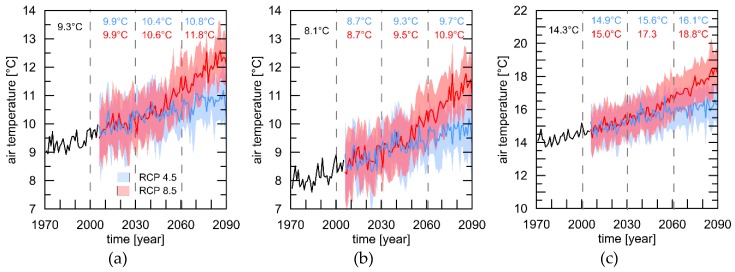
Multi-model ensemble of mean annual air temperature values for London (**a**), Luxembourg (**b**), and Rome (**c**) based on two different Representative Concentration Pathways (RCPs) (4.5 = blue; 8.5 = red). Spread is defined via +/− one standard deviation of the ensemble. Please note that the scale of Figure 1c is different from a and b.

First, we analysed the predicted change in annual air temperatures for the three different cities based on the multi-model ensemble and two different RCPs. Figure 1 highlights the results of the multi-model ensemble for mean annual air temperatures for London, Luxembourg, and Rome. The future climate projections of the nested regional climate model approaches show increases of the mean annual air temperatures for all three cities and the two different RCPs. London shows a 30-year mean of 9.3 °C for the reference period. An increase of the mean annual air temperature of 1.5 °C until end of this century is predicted for RCP4.5 and 2.5 °C for RCP8.5 (Figure 1a), respectively. With 8.1 °C, the reference period in Luxembourg is more than one degree colder than London but the projected increases for the RCPs are in comparable range at 1.6 °C (RCP4.5) and 2.8 °C (RCP8.5) (Figure 1b). Rome shows the highest increase from the reference period 1979–2000 (14.3 °C) to 2061–2090, with 1.8 °C (RCP4.5) and 4.5 °C (RCP8.5) (Figure 1c). All differences between the reference period and the three different future time spans for the three cities and two RCPs were statistically significant (*p* < 0.001).

**Figure 2:**
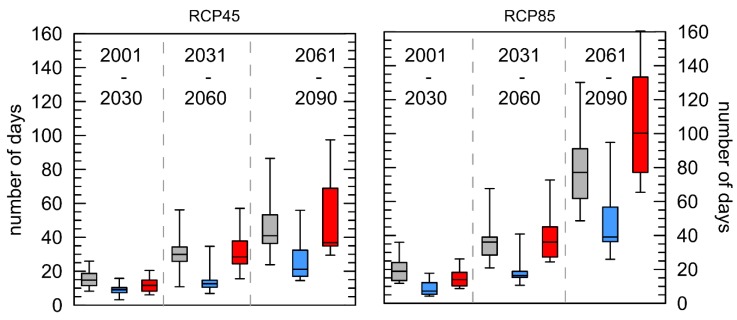
Boxplots of mean anomalies of the “Warm spell duration indicator” (WSDI) per year with reference to the 30-year period 1971–2000 for the three cities London (grey), Luxembourg (blue), and Rome (red) and two different RCPs. Whiskers = 5/95 percentile.

Figure 2 shows the boxplots of mean anomalies of the WSDI per year with reference to the 30-year period 1971–2000. The number of days contributing to events where the maximum air temperature is higher than the 90th percentile of the reference period on more than six consecutive days increases. Even though the mean annual air temperatures for London increases less in both RCPs than for Luxembourg, the corresponding WSDI exhibits stronger increases. 

Comparing the anomalies between the cities, there are significant differences between London/Luxembourg (both RCPs) and Rome/Luxembourg, for the near future (2001–2030). For the middle (2031–2060) and far future (2061–2090) statistically significant differences could be observed for Rome/Luxembourg and London/Luxembourg for both RCPs. Especially for Rome, the mean of the WSDI increased from 51 to 105 days (please note that the box plots show median values and not the arithmetic means).

**Figure 3:**
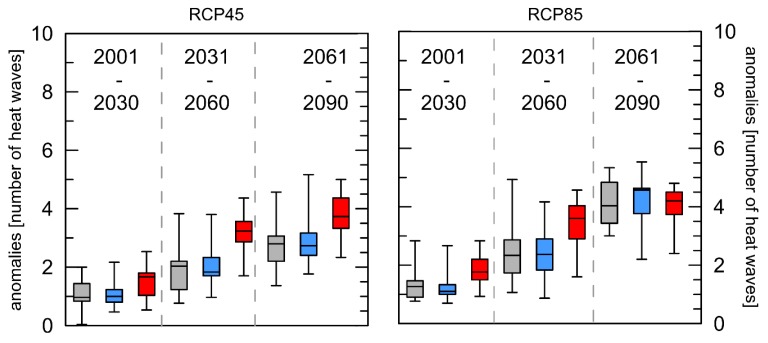
Boxplots of mean anomalies of the number of heat waves per year (HW-N) with reference to the 30-year period 1971–2000 for the three cities London (grey), Luxembourg (blue), and Rome (red) and two different RCPs. Whiskers = 5/95 percentile.

The following four indices are based on different definition of heat waves compared to the WSDI (description in Section 2.2). For the absolute number of HWs, all thirteen ensemble members show positive anomalies compared to their individual reference periods. The boxplots in Figure 3 illustrates that there are no big differences in the absolute number of HWs between RCP 4.5 and RCP 8.5. In both scenarios, there is an increase of one HW for London and Luxembourg for the near and two for the middle future. For the far future London and Luxembourg will have three more HWs for RCP 4.5 and four for RCP8.5. 

Differences between the cities are not significant for the near future for RCP4.5 and for the far future for RCP8.5. For all other time spans significant differences could be observed between Rome/London as well as Rome/Luxembourg for both RCPs.

**Figure 4:**
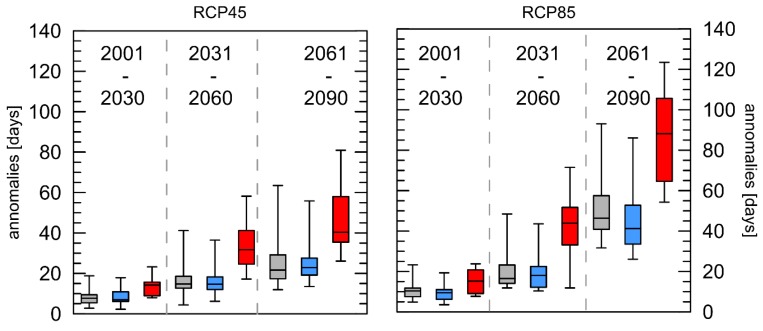
Boxplots of mean anomalies of the number of days’ contribution to heat waves each year (HW-F) with reference to the 30-year period 1971–2000 for the three cities London (grey), Luxembourg (blue), and Rome (red) and two different RCPs. Whiskers = 5/95 percentile.

The number of days that contribute to the absolute number of HWs were analysed further (Figure 4). For both RCPs in the near future, only slight changes could be observed. Already in the middle future, differences become obvious between London and Luxembourg compared to Rome, and this effect is even more pronounced for the far future. The mean number of days that contribute to an HW in Rome amounts to 47 (RCP4.5) and 86 (RCP8.5) in the far future time span. 

The statistical analysis of the differences between the cities showed that for all cases, the differences between Rome/London as well as Rome/Luxembourg are significant and, those between London/Luxembourg are not.

**Figure 5:**
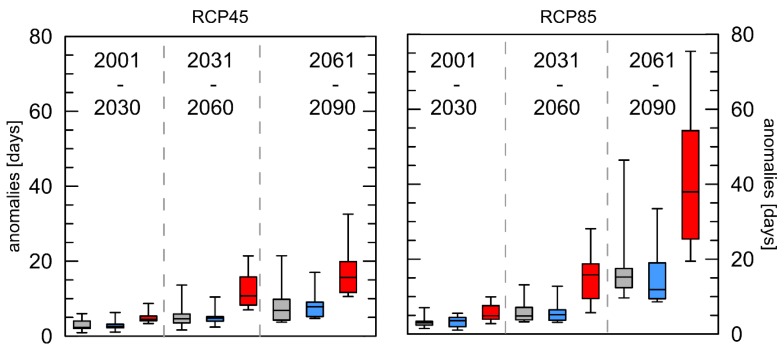
Boxplots of mean anomalies of the length of the longest heat waves each year (HW-D) with reference to the 30-year period 1971–2000 for the three cities London (grey), Luxembourg (blue), and Rome (red) and two different RCPs. Whiskers = 5/95 percentile.

A more detailed analysis, strongly related to health impacts, looks at the length of extreme heat events. Therefore, the anomalies for the length of the longest heat wave per year are shown in Figure 5. Again, a similar pattern can be observed with a strong increase for the far future only for Rome for RCP8.5. For London and Luxembourg, in the near future for both RCPs a prolongation of approximately 3 days is calculated, and for the middle future 5 and 6 days, respectively. Once more, the strongest increase is observed for Rome with a prolongation of 18 days for RCP4.5 and 41 days for RCP8.5.

The comparison between the cities yields the same significant differences between for Rome/London and Rome/Luxembourg, while the London/Luxembourg differences are not significant.

**Figure 6:**
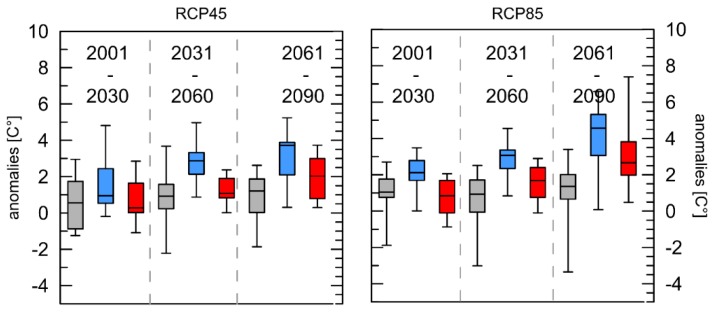
Boxplots of mean anomalies of the mean temperature of all heat waves per year (HW-M) with reference to the 30-year period 1971–2000 for the three cities London (grey), Luxembourg (blue), and Rome (red) and two different RCPs. Whiskers = 5/95 percentile.

The last index describes the mean air temperature of all detected HWs per year (Figure 6). In contrast to the others indices, here the results are less homogeneous. Especially for London, some of the ensemble members project even slight negative anomalies. However, overall a general increase for both RCPs in the mean air temperature during the heat wave can be observed for all cities and all future time spans. The differences for near future of the RCP4.5 scenario are not significant, those for the far future of the same RCPs are. For the middle future, only the differences between Rome/Luxembourg and Rome/London are significant. For the RCP8.5 in the near future the differences between Luxembourg/Rome and Luxembourg/London are significant, for the middle future all differences, and for the far future, Luxembourg/London and London/Rome.

## 4. Discussion

Climate warming will directly impact the probability of the occurrence of future extreme events and, thus, negatively impact different sectors like agriculture, ecosystems and human health [6,22]. Unfortunately, there is no standardized universal definition of HWs, meaning that direct comparisons between reported results are sometimes complicated, but the overall definition describes HWs as time spans of consecutive days with hotter conditions than usual [14]. To contribute to the unification efforts of the World Meteorological Organisation (WMO), the indices defined by the Expert Team on Sector-specific Climate Indices (ET-SCI) were used in this study. In general, past heat waves are a well investigated phenomena, e.g., the Chicago HW from 1995 [23,24] or the Paris HW from 2003 [25,26,27]. For the assessment of future frequencies and intensities of HWs, climate change projections are useful tools to derive potential future risks [5,28,29,30,31]. 

In our analysis, we rely on a multi-physics and multi-model ensemble of different regional climate models forced with two different emission scenarios (RCP4.5 and RCP8.5.). No weighting of the individual model results was used. To reduce the noise of the results, averages of 30-year time spans were calculated and it was these results that were finally used for the statistical analysis [29].

The projected changes in air temperature for the three different cities shown in Figure 1 are in line with results from comparable studies for London [32], Luxembourg [33,34,35,36] and Rome [37,38]. For London, an increase in the long-term annual mean air temperature by 1.5 °C for the RCP4.5 and 2.5 °C for the RCP8.5 is projected. Past research demonstrated that there is a clear link between increased mortality and increased air temperature and HWs [12] and heat-related diseases like respiratory and cardiovascular illnesses [39]. In addition, there is a strong relationship between increased air temperatures and amplified near surface ozone concentrations that reinforce the negative health effects [40]. According to recent studies in England and Wales the mortality increases by 2.1% for each 1 °C increase in air temperature above the 95th percentile of the average yearly temperature [32,39,41]. With 1.6 °C and 2.8 °C the projected increase in air temperature is a bit more pronounced for Luxembourg. The consequences for the regional fauna and flora were analysed in different climate change impact studies in the past [34,35,36,42,43]. For Rome the highest anomalies of up to 4.5 °C were projected corresponding to results shown for the Mediterranean region [29].

The duration of the heat wave expressed as WSDI, shown in Figure 2, can be used as a measure for how long the population is exposed to high levels of heat stress. A continuous increase in the WSDI could be observed for all cities with the highest anomalies for Rome and RCP8.5, which is consistent with the trends in the mean annual air temperature shown in Figure 1. Long-term, continuous exposure to high ambient air temperatures—targeted by this index—during the nights can decrease sleep quality significantly [44] especially for older people [45]. The WSDI was already applied for the past climate worldwide based on the HadEX2 data set [46]. In Europe for the time span 1961–2010, positive trends between 2.3 and 2.9 days per decade [47] could be observed. The impacts of projected changes in air temperature based on that index were analysed e.g., for China [22], showing a statistically significant increase of 2.4 days per decade on average since 1961. 

The index shown in Figure 3 comprises the absolute number of HWs per year (HW-N) and is strongly related to the index describing the number of days (Figure 4) that contribute to HWs per year (HW-F). The absolute number of days that contribute to HWs influences the number and the duration of the events but HW-F is not equal to HW-D multiplied with HW-N, because HW-D indicates the duration of the longest event, not the average duration [14].

Based on the “in the meantime updated SRES emission scenarios” an increase in the globally averaged (land only) number of HWs between 4 and 12 depending on the emission scenario was simulated [48]. Although there is not much difference in the number of heat waves between the two scenarios, the number of days’ contribution to heat waves each year with reference to the 30-year period 1971–2000 increases significantly following the projected general increase in air temperature. It is worth noticing, that the arithmetic mean of the HW-N anomalies for Rome in the RCP8.5 scenario for the far future is lower (+4.0 HW) than that for London (+4.1 HW) and Luxembourg (+4.2 HW) and additionally, the increase for the far future is less pronounced here than for the other indices. This could be explained by the fact that the increased number of days that contribute to HWs prolonged the length of the HWs substantially, so that few but very long HWs occur in all three cities every year at the end of this century. This is reconfirmed by the results presented in Figure 5. The HW-D index—describing the length of the longest HW per year—shows overall positive anomalies with the most pronounced positive trends for the far future. For both RCPs for Rome, the highest anomalies were projected for the far future. This index only describes the most extreme event per year, but is important for the health aspects because the longer the heat waves last, the more pronounced heat-related illnesses are. For Mumbai (India) an increase of one day in the duration of an HW was associated with a 4.9% increase in the estimated association between HWs and mortality [49]. The detected increase in the duration of heat waves in the future are similar to those presented for Eastern Europe or northern Russia [50].

Results of the analysis of the HW-M index also showed increases in the mean temperature during the HWs for all cities and RCPs, where Luxembourg showed the strongest increase (Figure 6). For England and Wales 1.0% of all summer deaths were attributable to air temperature [51], and an increase of 1 °C corresponded to a 3.1% increase in daily mortality. In Mediterranean cities a 2% increase in mortality is associated with a rise of approximately 10 °C [13].

Further research is necessary to analyse the timing of the HWs within a year, because HWs occurring earlier in the summer may have greater effects on mortality [52,53]. The importance of the age structure is highlighted by the 2003 HW in France. Beginning of August 2003 air temperatures were above 35 °C, and at some meteorological stations even above 40 °C [54]. More than 15,000 excess deaths were observed [55]. It was shown by [56] that mortality excess was estimated to 20% (age 45–74 years), 70% (age 75–94 year) and 120% (older than 94 years). This was also shown for other European countries by for different age classes [57].

Beside the effects of HWs on health they also might negatively influence food security and water supply, leading to higher incidence of water-borne infectious and toxin-related illnesses, such as cholera or bluetongue emergence [58,59]. Furthermore, HWs are of highly relevance for hydrological processes but also agriculture and forestry [60,61,62,63].

## 5. Conclusions

To understand projected changes in the frequency and intensity of HWs and their potential negative impacts on public health a detailed knowledge of the projected changes in air temperature distribution is fundamental [64]. A consistent increase in the frequency and intensity of HWs for the three selected cities was detected by the use of common climate change indices, recommended by the WMO and is in line with comparable results from different working groups [65,66,67]. Even if anthropogenic greenhouse gas emissions could be completely stopped, a continuous warming will take place over the next few decades.

The influence of elevated heat stress on public health has been demonstrated in past studies. A better understanding of the health risks and health impacts of extreme events, combined with the knowledge of the expected changes for dedicated cities, is a prerequisite for further steps. Beside the necessary mitigation efforts, that must be undertaken to limit climate warming to a minimum, increased resilience and the formulation of appropriate adaptation measures is crucial to reducing negative health impacts in the future. Additionally, a science-based prioritisation of urban planning measures, implementation of heat warning systems and other effective public health actions will be possible based on such analysis. 

## Figures and Tables

**Table 1:** Regional climate change projection datasets used in the study. The table contain the model abbreviations used in the text, the driving Global Climate Model (GCM), the Regional Climate Model (RCM) used for the dynamical downscaling as well as the institutions that are responsible for the model runs; temporal resolution: daily data; time span: 1971–2091.

Model Number	Official CMIP5 GCM Name	Modelling Centre/Group CMIP5 “Institute ID”	Official EURO-CORDEX RCM Name	Modelling Centre/Group EURO-CORDEX “Institute ID”
M01	CNRM-CM5	CNRM-CERFACS	ALADIN53	CNRM
M02	CNRM-CM5	CNRM-CERFACS	ALARO-0	RMIB-UGent
M05	MPI-ESM-LR	MPI-M	REMO2009	MPI-CSC
M06	MPI-ESM-LR	MPI-M	RCA4	SMHI
M07	NorESM1-M	NCC	HIRHAM5	DMI
M09	HadGEM2-ES	CNRM MOHC	RCA4	SMHI
M10	IPSL-CM5A-MR	IPSL	WRF331F	IPSL-INERIS
M11	CNRM-CM5	CNRM-CERFACS	CCLM4-8-17	CLMcom
M12	EC-EARTH	ICHEC	RACMO22E	KNMI
M13	IPSL-CM5A-MR	IPSL	RCA4	SMHI
M14	MPI-ESM-LR	MPI-M	CCLM4-8-17	CLMcom

**Table 2:** Definition of extreme temperature indices according to ClimPact2.

Abbreviation	Definition	Unit
HW-D	Length of longest HW	days
HW-F	Number of days that contribute to heat waves	days
HW-M	Mean temperature of all heat wave in a year	°C
HW-N	Number of heat waves per year	number
WSDI	Number of days contributing to events where on > 6 consecutive days the maximum air temperature is in > 90th percentile	days
